# Selecting Aragonez Genotypes Able to Outplay Climate Change–Driven Abiotic Stress

**DOI:** 10.3389/fpls.2020.599230

**Published:** 2020-12-17

**Authors:** Luísa Carvalho, Elsa Gonçalves, Sara Amâncio, Antero Martins

**Affiliations:** LEAF- Linking Landscape, Environment, Agriculture and Food, Instituto Superior de Agronomia, Universidade de Lisboa, Lisbon, Portugal

**Keywords:** abiotic stress tolerance, empirical best linear unbiased predictors (EBLUP of genotypic effects), polyclonal selection, quality of the must, RT-qPCR

## Abstract

High temperatures and extreme drought are increasingly more frequent in Portugal, which represents a strong threat to viticulture in certain regions of the country. These multifactorial abiotic stresses are threatening viticultural areas worldwide, and the problem can hardly be overcome only by changing cultural practices. This scenario has raised a major challenge for plant scientists to find ways to adapt existing varieties to the new conditions without loss of their characteristic flavors, yield, and associated varietal character of wines. Aragonez (syn. Tempranillo) is one such variety, widely cultivated in Portugal and Spain, with specific characteristics associated with *terroir*. In this context, insight into intravarietal variability to enable its exploitation for selection becomes an important tool to mitigate the effect of multifactorial stresses driven by climate changes. The present work describes an innovative selection approach: selection for abiotic stress tolerance, measured by the leaf temperature of clones under environmental conditions of drought and extreme heat. This evaluation was complemented with values of yield and quality characteristics of the must (pH, acidity, °Brix, and anthocyanins). The application of this methodology was done in an experimental population of 255 clones of Tempranillo for 3 years. The genotypes were then ranked according to their level of tolerance to abiotic stress without loss of yield/quality. To understand the differences at the transcription level that could account for such variability, several of the most tolerant and most sensitive genotypes were analyzed for key genes using reverse transcriptase–quantitative polymerase chain reaction. The results enabled the selection of a group of genotypes with increased tolerance to stress, in relation to the average of the variety, which maintained the typical must quality of Aragonez. In parallel, several transcripts previously acknowledged as markers for abiotic stress tolerance were identified in several clones and are possible targets for plant breeding and genetic modification and/or to develop screening procedures to select genotypes better adapted to the abiotic stress driven by climate change.

## Introduction

Grapevine is one of the most economically important crop species in Portugal. Its main product, grapes, feeds the wine sector, highly significant for the national economy and a major export. In 2018, Portugal was the fifth wine producer in the European Union, and the 11th worldwide (International Organisation of Vine and Wine [OIV], 2018). Viticulture is highly dependent upon climatic conditions during the growing season, and thus wine production is being affected by climate change. Return on investment is driven by yield and its quality; thus, it is relevant to study the impact of climate change, namely, the implications of changes in temperature levels and patterns, radiation, and water availability on those parameters. Portugal is extremely rich in autochthonous grapevine varieties with more than 250 already known and with a high level of intravarietal variability (Martins and Gonçalves, 2015).

Intravarietal diversity has ensured stable behavior of the varieties over time and constitutes today the raw material for carrying out the selection with high genetic and economic gains of different relevant characteristics. Polyclonal selection consists in the selection of a top-ranked set of genotypes concerning target traits in an experimental population containing a representative sample of the intravarietal variability, and the genetic gains of selection are predicted as the average of the empirical best linear unbiased predictors (EBLUPs) of the genotypic effects of those traits in the set of selected genotypes (Martins and Gonçalves, 2015). Theoretical developments about the most suitable experimental designs and statistical models for the quantification of intravarietal diversity and prediction of genetic gains of polyclonal selection have been developed in the past years (Gonçalves et al., 2010, 2013). Specifically for selection purposes, the multivariate mixed-model approach proposed by Gonçalves et al. (2016) was shown to provide greater accuracy and precision in selection. Actually, the exploitation of intravarietal genetic variability became a crucial strategy to face future challenges (climate change, biotic and abiotic stresses, consumer demands, etc.), and its importance was recently recognized by the OIV through the adoption of Resolution OIV-VITI 564B-2019 (2019).

Vine phenology, that is, the date on which bud break, flowering, *véraison*, and maturation occur, is driven by temperature. This relation is so strong that vine phenology can be predicted by models based on temperature alone (Parker et al., 2011). During ripening, sugar accumulation increases with temperature, but certain secondary metabolites, such as anthocyanins, are negatively affected by high temperature (van Leeuwen and Darriet, 2016). Grape acidity, in particular, malic acid content, decreases in high temperature (van Leeuwen and Darriet, 2016).

Limited vine water availability affects photosynthesis and leaf transpiration (Chaves et al., 2010), shoot (Carvalho et al., 2016), and root development (Dry et al., 2000), and leaf and berry mineral nutrition (Gonzalez-Dugo et al., 2010). Stomatal control of leaf transpiration is a physiological drought avoiding mechanism that enables the optimization of crop water use while preventing embolism events (Lovisolo et al., 2010). The severity of a drought and its timing and duration impair final berry size and composition (Deloire et al., 2004). Generally, moderate water stress during the ripening period is favorable for sugar accumulation (van Leeuwen et al., 2009) and increases the anthocyanin and tannin contents in berries (van Leeuwen and Darriet, 2016). In temperate climates, conditions of water deficit are favorable for producing high-quality red wines (van Leeuwen et al., 2009).

Grapevine varieties demonstrate significant variability in their hydraulic behavior (Schultz, 2003), a feature reflected in the variety-specific responses to water deficit (Carvalho et al., 2016). Differences in drought tolerance between varieties (Carvalho et al., 2016; Carvalho et al., 2017) are likely due to differences in root-to-shoot signaling and differential hydraulic regulation between those varieties (Vandeleur et al., 2009; Zarrouk et al., 2016).

In the wine industry, the distinct and recognizable characteristics of wine are usually attributed to differences in the chemical composition of flavonoid compounds, as a varietal “signature” (Downey et al., 2006). Phenylalanine-derived polyphenols accumulate mainly in the skin of grape berries, during the course of fruit development, and comprise mainly flavonols, flavan-3-ols (flavanols), and anthocyanins (Downey et al., 2006). Their accumulation in berries varies among varieties, developmental stages, growing regions, and viticultural practices in relation to irradiation, nutrient, and temperature changes (Downey et al., 2006). In fact, there is differential regulation in the berry polyphenol metabolism in drought-prone environments (Degu et al., 2014). Nevertheless, flavonoid synthesis and degradation in response to heat waves and in respect to intravarietal variability are yet to be elucidated. Identifying genotypes (clones) showing higher stress tolerance or better performance under high temperatures and drought is a major objective of present-day grapevine selection.

As a reference variety for the intravarietal diversity analysis, we selected a major wine variety in Portugal, known as Aragonez in Alentejo and Tinta Roriz in Douro and internationally known as Tempranillo. Vegetative multiplication of this variety for centuries has originated the accumulation of somatic mutations that have been the base for its adaptation to current growing conditions in different wine-making regions of the world and could also be useful for adaptation to climate change. Any genetic variation identified in screenings for abiotic stress could immediately be used to select clones with improved stress adaptive traits.

An innovative approach was chosen because of the importance of developing a method to quantify the plant’s response to changes in the environment that can be expedited and is reproducible and non-invasive, to accurately scan a large population in real time. Surface leaf temperature (SLT), measured with a portable infrared thermometer, is a parameter that sets the boundary condition for the latent and sensible heat transport through vegetation, soil, and atmosphere, depending on the availability of moisture at the interface soil atmosphere (Fuchs, 1990), giving an estimate of the response of a leaf to the environmental parameters affecting it at any time (air temperature, relative humidity, solar radiation, leaf resistance, and boundary layer resistance) (Udompetaikul et al., 2011). By utilizing appropriate measurement devices, the relationship between these parameters can be studied. A plant is able to keep an SLT lower than ambient temperature by controlling stomatal aperture and thus gas and water vapor exchanges through stomata. The capacity to control stomata opening and thus CO_2_ intake for photosynthesis regardless of high air temperature allows identifying the clones tolerant to face impending climate change without loss of productivity and quality of their grapes.

In previous works, we thoroughly characterized the different and contrasting responses of the varieties Touriga Nacional (TN) and Trincadeira (TR) to abiotic stresses (Carvalho et al., 2015a,b; Rocheta et al., 2016), showing that TN is a variety that can withstand severe levels of stress without being much affected, whereas TR is more sensitive. We have also proven that 49 of the DEGs (differentially expressed genes) identified by Rocheta et al. (2016) can be used as “abiotic stress markers” to characterize stress tolerance of grapevine varieties and as indicators of the major kind of stress the plant is subjected to (drought, heat, or excess light) (Carvalho et al., 2017). With the objective of studying the variability of tolerance to abiotic stress within varieties, we chose the red variety Aragonez (syn. Tempranillo), a variety that is known to have high variability regarding yield and quality traits (Gonçalves et al., 2007; Gonçalves and Martins, 2019).

Under the hypothesis that abiotic stress-tolerant clones show lower average SLT, a large collection of Aragonez clones in a field trial was evaluated. In this selection assay, established in Alentejo, in Reguengos de Monsaraz, 255 genotypes of this variety were used for stress tolerance monitoring based on the identification of clones with lower SLT together with analyses of berry trait variation. SLT was used as a non-invasive and expedite indicator of abiotic stress tolerance. These clones were subjected to a detailed analysis during 2014, 2015, and 2016 seasons to identify clones tolerant to stress, regardless of environmental conditions, and among those, the ones that gave rise to musts with good-quality traits for wine production. Taking advantage of this analysis, randomly chosen clones from the tolerant and sensitive ranking groups were scanned using quantitative polymerase chain reaction (qPCR) for the expression of the transcripts previously identified.

## Materials and Methods

### Experimental Design and Location of the Field Trial

The evaluations were performed in an experimental population of clones of the Aragonez variety, containing representative samples of the intravarietal diversity in different growing regions of Portugal (Alentejo and Douro) and Spain (Rioja and Valdepeñas). This field trial is located in Reguengos de Monsaraz (Alentejo, Portugal) and was established in 1996, according to a balanced randomized complete block design (255 genotypes × three plants per plot × five blocks). As control of heterogeneity within complete blocks is best accomplished with a row–column arrangement within each complete block, the plots (experimental unit with three plants) were located on a grid of columns by rows. All plants were grafted on the same clone of 1103P rootstock and were free from grapevine leafroll associated virus type 3 and grapevine fanleaf virus. The training system was a vertical shoot position, and the pruning system was a bilateral Royat Cordon system.

### Abiotic Stress and Quality Traits Evaluation

The evaluations were conducted in 2014, 2015, and 2016 seasons. The field was drip irrigated, but *circa* 2 weeks before the first SLT quantifications, irrigation was upheld and resumed only after the measurements were finished. Water stress conditions were quantified through the measurement of predawn leaf water potential (pressure chamber; Model 600, PMS Instruments Company, Albany, OR, United States) in the field. The average values obtained were of moderate stress in 2014 (−0.55 MPa) and severe stress in 2015 (−0.73 MPa) and 2016 (−0.7 MPa).

For SLT evaluation, the original experimental design was updated to control as strictly as possible the environmental effects (namely, the effects of the day and time of the evaluation). Each complete block was evaluated per day and in a day the plants of the plots of each column were measured in the shortest time possible. As a consequence, a resolvable incomplete block experimental design was adopted: (1) each complete block comprised the effect of the original experimental design and the effect of the day; (2) each column within each complete block, with approximately 13 plots, constituted an incomplete block, which comprised the effect of the time of day. In each plot, three measurements were performed in three different leaves with 10 technical replicates. Measurements were taken on peak heat hours on leaves exposed to the sun using a non-contact IR thermometer (Scan Temp 440).

Berry quality traits (soluble solids, acidity, pH, anthocyanins, and total phenols) were analyzed in the must, as well as berry weight. Berry collection was performed for all genotypes in three complete blocks. A sample of 60 berries per plot (experimental unit) was collected the day before the harvest. In laboratory, the berries from each plot were counted and weighted, and grape must was obtained from berries by applying the sample preparation procedure described by Carbonneau and Champagnol (1993). The analyses of the must were performed by standard methods: soluble solids by refractometry (probable alcohol by conversion), acidity by titration, and anthocyanins and total phenolics by spectrophotometric method described by Somers and Evans (1977).

### Data Analysis

A preliminary univariate analysis for each trait and year was conducted to verify the quality of the data obtained in each year and the existence of significant genetic variability (*P* < 0.05) for the studied traits. For the analysis of SLT data, a linear mixed model was fitted, considering fixed effects for complete blocks and random effects for genotype, incomplete block within replicate (complete block), and leaf (within plot). For data analysis of berry weight and quality traits of the must, a linear mixed model was fitted for each trait, considering fixed effects for complete blocks and random effects for genotype. In these models, random effects and random error were assumed independent and identically distributed normal random variables with expected value zero and the respective variance.

The variance parameters were estimated by the restricted maximum likelihood method (Patterson and Thompson, 1971) using the average information algorithm (Gilmour et al., 1995). The variance components were tested using a residual maximum likelihood ratio test. Because the null hypothesis was on the boundary of the parameter space, the *P*-value of the test was assumed to be half of the reported *P*-value from the χ^2^ distribution with one degree of freedom (Self and Liang, 1987; Stram and Lee, 1994).

For selection purposes, the multivariate mixed-model approach proposed by Gonçalves et al. (2016) was adopted. For SLT, three response variables (“traits”) were considered (SLT2014, SLT2015, and SLT2016); for berry traits, for each year, six response variables were included (soluble solids, pH, acidity, anthocyanins, total phenols, and berry weight). For both analyses, an unstructured covariance matrix was assumed between traits. From this methodology, quantitative genetic analysis was implemented through the indicators described as follows: (1) a generalized measure of heritability for each trait was obtained, based on prediction error variance and genotypic variance component estimates (Gonçalves et al., 2013); (2) for each multivariate model, genetic correlations between pairs of traits were obtained; (3) the EBLUPs of the genotypic effects of the studied traits were obtained through mixed-model equations (Henderson, 1975); the predicted genotypic values (PGVs) were computed, and both were ranked to characterize the more tolerant genotypes (rank for SLT) and for quality traits of the berries; (4) for each year, the Pearson correlation coefficient between the PGVs of SLT and each berry trait was calculated.

Data analysis was carried out with R (R Core Team, 2018; The R Foundation for Statistical Computing Platform). Linear mixed models were fitted using ASREML-R software (Butler et al., 2018).

### Polyclonal Selection

The EBLUPs of genotypic effects for the studied traits provide information about the genetic component affecting those traits, and selection should be performed based on those values. Thus, the EBLUPs of the genotypic effects of the studied traits and the PGVs were ranked to characterize the more tolerant genotypes (rank for SLT). A set of 12 genotypes out of the 30 with lower SLT and that are present simultaneously in 2 or 3 years of evaluation were selected (when present simultaneously only in 2 years, in the third year, the genotype was, in the worst scenario, on the average, i.e., with EBLUP of genotypic effect near zero). Their behavior for the other evaluated traits was then analyzed. The yield data obtained in previous evaluations (Gonçalves et al., 2007) were also considered for the characterization of the final selected group. The predicted genetic gains for SLT, quality traits, and yield for the group of tolerant genotypes were computed as the average of the EBLUPs of the genotypic effects of each of the traits in the selected group of genotypes.

### RNA Extraction

Samples were collected in 2015, under the same conditions as the measurements of SLT, and kept at −80°C. For gene expression analysis, three replicates of five clones within the best ranking clones for SLT in 2014 and five clones within the worst ranking ones in the same year were used. Samples were ground in the presence of liquid nitrogen with a mortar and pestle. Total RNA was extracted with the Spectrum^TM^ Plant Total RNA kit (Sigma–Aldrich, St. Louis, MO, United States). In all samples, nucleic acid concentration was quantified by spectrophotometry using the software Gen5 1.09 (Synergy HT; BioTek Instruments, Winooski, VT, United States). The quality of the extracted RNA was evaluated using A260/A280 and A260/A230. To be used, samples had to have ratios A260/A280 between 1.8 and 2.1 and A260/A230 between 2.0 and 2.2. Total RNA integrity was assessed through 1% agarose gel electrophoresis under denaturing conditions.

### cDNA Synthesis for qPCR

RNA samples were treated with RQ1 RNase-Free DNase (Promega, Madison, WI, United States). cDNA was synthesized from 2 μg of total RNA using oligo(dT)_20_ in a 20-μL reaction volume using RevertAid Reverse Transcriptase (Fermentas Life Science, Helsingborg, Sweden) according to the manufacturer’s recommendations. cDNA was tested for gDNA contamination in PCRs using intron spanning primers that yield a 229-bp amplicon in cDNA and a 547 amplicon in gDNA. Amplicon sizes were compared in 2% agarose gels together with the molecular weight marker 1 Kb+ (Invitrogen), and no gDNA contamination was detected. cDNA was stored at −20°C until further use.

### Quantitative Polymerase Chain Reaction (qPCR)

Primers were designed using the software Beacon Designer (Premier Biosoft) using a primer length of 20 ± 2 bp, melting temperature of 60°C ± 2°C, a guanine–cytosine content of circa 50% and an expected amplicon size of 180–280 bp. Sequences were the same as in Carvalho et al. (2017). The real-time qPCR was performed in 96-well white reaction plates (Bio-Rad, Hercules, CA, United States), using an IQ5 Real Time PCR (Bio-Rad) with five biological replicates. The 20-μL reaction mixture was composed of 1 μL cDNA diluted 50-fold, 0.5 μM of each gene-specific primer, and 10 μL master mix (SsoFast_EvaGreen Supermix; Bio-Rad). Amplification of PCR products was monitored via intercalation of Eva-Green (included in the master mix). The following program was applied: initial polymerase activation, 95°C, 3 min, and then 40 cycles at 94°C 10 s (denaturation), 60°C 20 s (annealing), and 72°C 15 s (extension), followed by a melting curve analysis to confirm the correct amplification of target gene fragments and the lack of primer dimers. The PCR products were run on 2% agarose gels to make sure that there was only one amplicon of the expected size. PCRs with each primer pair were also performed on samples lacking cDNA template, in triplicate (no template controls). To assess amplification efficiency of the candidate genes, identical volumes of cDNA samples were diluted and used to generate five-point standard curves based on a fivefold dilution series (1, 1:5, 1:25, 1:125, 1:625), in triplicate. Amplification efficiency (E) is calculated as *E* = 10(−1/a) − 1, “a” being the slope of the linear regression curve [*y* = *a* log(*x*) + *b*] fitted over the log-transformed data of the input cDNA dilution (*y*) plotted against the respective quantification cycle (*C*q) values (*x*). *E*-values of the target genes were considered comparable when they did not exceed 100 ± 10%, corresponding to a standard curve slope of 3.3 ± 0.33. All cDNA samples were diluted 50-fold and were amplified in duplicate in two independent PCR runs.

To generate a baseline-subtracted plot of the logarithmic increase in fluorescence signal (ΔRn) versus cycle number, baseline data were collected between cycles 5 and 17. All amplification plots were analyzed with an Rn threshold of 0.2 at the beginning of the region of exponential amplification, to obtain *C*q (quantification cycle), and the data obtained were exported into an MS Excel workbook (Microsoft Inc., United States) for analysis. Reference genes used were ACT, TIF, and TIF-GTP (Coito et al., 2012).

### Statistical Analysis of Gene Expression

For the relation between the expressions of the selected genes and the reference genes, the relative quantity values were transformed into log2 (thus rendering them parametric) and tested through analysis of variance (ANOVA) in the program SAS 9 (for Windows; SAS Institute Inc., Cary, NC, United States). When the *P*-value of the ANOVA was lower than 0.05, a Tukey test was performed, and statistically significant differences were accepted for *P* < 0.05.

## Results

### SLT Analysis

The quantification of genetic variability within the variety for each trait was assessed by the estimate of the genetic variance component. From the results obtained from the univariate analysis (Table 1), it was possible to verify that there is significant genetic variability within the variety for the trait SLT (*P* < 0.001). The other variance components associated with the experimental design were all significant (*P* < 0.001), as well as the fixed-effects factor (complete block).

**TABLE 1:** Variance components estimates and respective standard errors, obtained with the fitting of the univariate linear mixed model for SLT in 2014, 2015, and 2016, and the *P-*value of the likelihood ratio test for variance components (testing the null hypothesis if the variance component is zero).

**Year**	**Variance component**	**Estimate (*SE*)**	***P***
2014	Genotypic	0.664 (0.108)	<0.001
	Incomplete block within complete block	6.157 (0.970)	<0.001
	Leaf (within plot)	7.034 (0.176)	<0.001
	Error	0.606 (0.005)	
2015	Genotypic	0.725 (0.104)	<0.001
	Incomplete block within complete block	6.910 (1.081)	<0.001
	Leaf (within plot)	5.609 (0.140)	<0.001
	Error	0.441 (0.003)	
2016	Genotypic	0.766 (0.115)	<0.001
	Incomplete block within complete block	4.308 (0.687)	<0.001
	Leaf (within plot)	6.623 (0.165)	<0.001
	Error	0.365 (0.003)	

*For the fixed-effects factor of the experimental design (complete block), the effect was significant in the 3 years (P < 0.01)*.

Empirical best linear unbiased predictors (EBLUPs) of genotypic effects and PGVs for SLT in 2014, 2015, and 2016 for all studied genotypes are provided in Supplementary Tables 1, 2, respectively, as well as the rank for tolerance to abiotic stress. The differences between the EBLUP of genotypic effect of the more and the less tolerant clones and the PGVs for SLT in 2014, 2015, and 2016 are shown in Table 2. On average, there is a genetic quantifiable difference of 4°C between the coolest and warmest of the 255 clones measured in each of the three consecutive seasons. The values obtained for generalized broad sense heritability were moderate (ranging between 0.44 and 0.54), but very interesting given the nature of the trait assessed. This quantitative genetic parameter is particularly useful to complement the genetic analysis as it reflects the relationship between the true and predicted genotypic effects and, consequently, is an indicator of the success of genetic selection. Genetic correlations for SLT between the 3 years were low, which indicates the existence of genotype × environment interaction. That is, the rank of the most tolerant clones changed over the years, although it was possible to select a group of clones consistently identified as the most tolerant in all evaluated seasons.

**TABLE 2:** Differences between empirical best linear unbiased predictors (EBLUPs) of genotypic effects for SLT between the most sensitive and most tolerant genotypes and the predicted genotypic value (PGV) of the more and the less tolerant clones for SLT in 2014, 2015, and 2016; broad sense heritability; and genetic correlations for SLT between the years (and respective standard error, SE), obtained with the fitting of the multivariate linear mixed model.

**Year**	**EBLUPs of genotypic effects for SLT**		**PGV for SLT**		**Broad sense heritability**	**Genetic correlation estimate (SE)**	
	**Most sensitive**	**Most tolerant**	**Most sensitive**	**Most tolerant**			
2014	+1.7°C	−1.5°C	33.3°C	30.1°C	0.44	SLT2014, SLT2015	0.084 (0.108)
2015	+2.3°C	−1.8°C	36.3°C	32.2°C	0.52	SLT2015, SLT2016	0.239 (0.100)
2016	+2.3°C	−2.2°C	34.1°C	29.6°C	0.54	SLT2014, SLT2016	0.285 (0.104)

### Must Quality Versus SLT Analysis

The results of the characterization of must quality traits within the Aragonez collection are shown in Table 3. These traits also showed the existence of significant genetic variability (for any usual significance level). For acidity, the values obtained for heritability were lower, and for anthocyanins, total phenols, and berry weight, they were similar to those obtained for SLT. Genetic correlations estimates between traits obtained with the fitting of the multivariate linear mixed model for berry traits in 2014, 2015, and 2016 were low, except the correlation between anthocyanins and total phenols in 2015 (Supplementary Table 3).

**TABLE 3:** Genotypic variance component estimates and respective standard errors, obtained with the fitting of the univariate linear mixed model for berry traits in 2014, 2015, and 2016, and average values of all seasons, the *P*-value of the likelihood ratio test for genotypic variance component, and broad sense heritability for all traits in all years obtained with the fitting of the multivariate linear mixed model.

**Trait**	**Year**	**Overall mean**	**Genotypic variance component estimate (*SE*)**	***P***	**Broad sense heritability**
Soluble solids (°Brix)	2014	25.76	1.788 (0.249)	<0.001	0.663
	2015	22.54	1.064 (0.171)	<0.001	0.615
	2016	22.07	0.725 (0.159)	<0.001	0.453
	Average	23.47	0.831 (0.106)	<0.001	0.710
pH	2014	4.25	0.007 (0.001)	<0.001	0.568
	2015	4.36	0.008 (0.002)	<0.001	0.543
	2016	4.36	0.004 (0.001)	<0.001	0.513
	Average	4.32	0.005 (0.001)	<0.001	0.644
Acidity (tartaric acid, g L^–1^)	2014	2.68	0.025 (0.008)	<0.001	0.307
	2015	2.71	0.010 (0.004)	0.003	0.299
	2016	2.43	0.013 (0.005)	0.003	0.262
	Average	2.61	0.009 (0.002)	<0.001	0.380
Anthocyanins (mg L^–1^)	2015	443.14	4439.622 (976.912)	<0.001	0.473
	2016	425.26	4385.909 (732.043)	<0.001	0.573
	Average	434.31	2754.188 (497.936)	<0.001	0.511
Total phenols	2015	34.69	37.646 (8.351)	<0.001	0.462
	2016	43.67	43.230 (8.107)	<0.001	0.514
	Average	39.21	25.169 (4.784)	<0.001	0.476
Berry weight (g)	2015	1.29	0.016 (0.004)	<0.001	0.455
	2016	1.33	0.008 (0.002)	<0.001	0.455
	Average	1.31	0.009 (0.002)	<0.001	0.417

For each year, genetic correlations between PGV of SLT and berry traits are described in Table 4. All the correlations were not significantly different from zero. This means that by exploring the genetic variability within the variety, several genotypes satisfying several criteria can be selected. For example, it is possible to select genotypes that are simultaneously more tolerant to stress and with berry traits above or near the mean of the population studied.

**TABLE 4:** Pearson correlation coefficient between the predicted genotypic values (PGVs) of SLT and each berry trait in 2014, 2015, and 2016.

**Traits**	**Year**	**Pearson correlation coefficient^†^**
SLT/°Brix	2014	0.034
	2015	–0.008
	2016	–0.114
SLT/pH	2014	0.120
	2015	–0.023
	2016	0.052
SLT/acidity	2014	–0.033
	2015	0.077
	2016	0.030
SLT/anthocyanins	2015	0.003
	2016	–0.009
SLT/total phenols	2015	0.022
	2016	–0.004
SLT/berry weight	2015	0.030
	2016	–0.042

*^†^Non-significant for all cases (P > 0.05)*.

For the polyclonal selection, the 255 genotypes were then ranked according to the EBLUP genotypic effects and PGVs for SLT. Additionally, the results of the average values of all years for berry traits were used (those with higher values of heritability). EBLUPs of genotypic effects and PGVs for berry traits for all studied genotypes are provided in Supplementary Tables 4, 5, respectively, as well as the rank for each trait. A set of 12 genotypes out of the 30 with lower SLT present simultaneously in 2 or 3 years of evaluation were selected. The features of selected polyclonal group are described in Tables 5, 6. Compared to the mean of the population, on average the SLT of the selected group decreased 1°C, corresponding to a predicted genetic gain of about 3% (as percentage of the mean of the population) in decrease of SLT. It must be emphasized that, on average, there is a genetic quantifiable difference of 3°C between this tolerant selected group and the highest value measured among the 255 clones in each of the three seasons (Table 5).

**TABLE 5:** Characterization of the selected group of 12 genotypes (polyclonal selection) for abiotic stress.

**Year**	**Predicted genotypic value for SLT**	**Decreasing in SLT comparing to the mean of the population**	**Predicted genetic gain (as percentage of the mean of the population)**	**Decreasing in SLT comparing to the most sensitive genotype**
2014	30.6°C	−1.0°C	−3.2%	−2.7°C
2015	33.1°C	−0.9°C	−2.6%	−3.2°C
2016	30.8°C	−1.0°C	−3.2%	−3.3°C

**TABLE 6:** Predicted genetic gains of the selected group of 12 genotypes (polyclonal selection) for abiotic stress concerning other traits.

**Trait**	**Predicted genetic gain (as percentage of the mean of the population)**
Soluble solids (°Brix)	0.0%
pH	−0.2%
Acidity (tartaric acid, g L^–1^)	+0.5%
Anthocyanins (mg L^–1^)	+0.7%
Total phenols	−0.4%
Berry weight (g)	0.0%
Yield (kg plant^–1^)	+14.1%

The behavior of the selected group for the other evaluated traits is shown in Table 6. The yield data obtained in previous evaluations were also considered for the characterization of the final selected group. A group of genotypes simultaneously tolerant and with higher yield was selected, ensuring a mean overall performance for berry traits. In fact, the predicted genetic gains for quality traits and berry weight were around zero, which means that, for the considered traits, the behavior of the selected group is around the mean of the variety. However, as a predicted genetic gain of yield of +14.1% was observed, a tendency of more tolerant genotypes to show a yield performance above the mean of the population can be considered.

According to the results obtained in 2014 for SLT (Supplementary Tables 1, 2), 10 genotypes were selected for gene expression analysis: five tolerant (RZ1124, RZ1338, RZ6210, RZ1502, and RZ1703) and five sensitive (RZ0703, RZ1243, RZ8207, RZ3710, and RZ3408).

### Abiotic Stress Array in the 10 Tolerant and Sensitive Clones

As indicated above, for gene expression analysis clones were chosen as sensitive or tolerant according to SLT results of 2014. However, after the global 3-year PGV of SLT analysis, a third group emerged of clones highly affected by genotype × environment interaction, thus changing their rank each season. These were kept in the analysis, as it is also important to identify these highly variable genotypes. Thus, the three-group analysis comprised as tolerant, the clones RZ1124, RZ1338, and RZ6210; as sensitive, the clones RZ3408 and RZ3710; as variable, the clones RZ0703, RZ1243 RZ1502, RZ1703, and RZ8207. As samples for gene expression were taken in 2015, comparisons with SLT were made using values of that same season.

**FIGURE 1:**
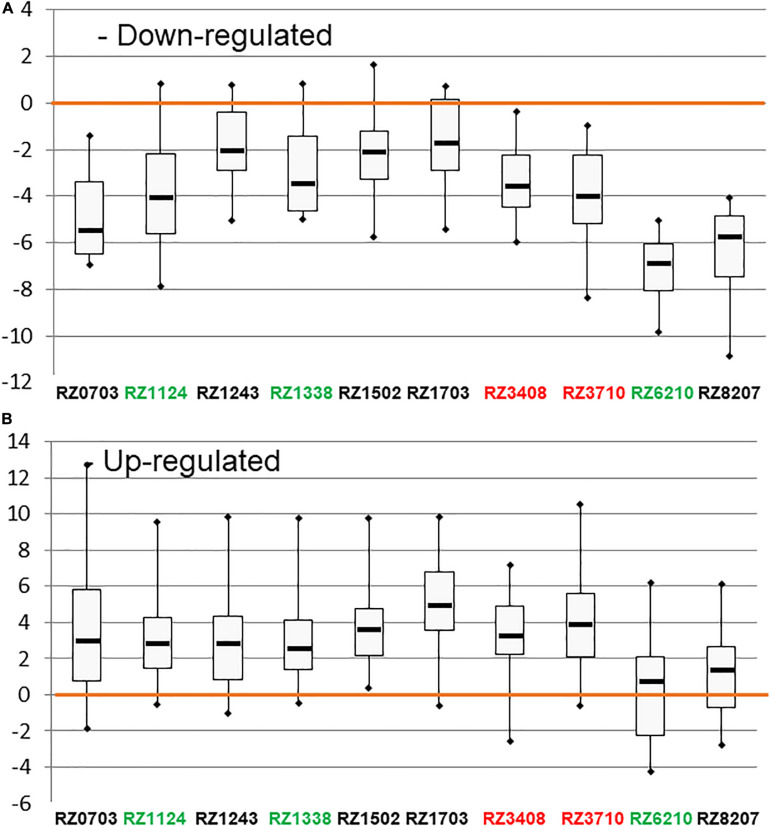
Box plots representing the distribution of the expression of down-regulated **(A)** and up-regulated **(B)** DEGs (as in Supplementary Table 6) in [log_2__(gene expression ratio)_] in the 10 chosen Aragonez clones. The color code of the varieties’ names, according to the overall SLT analysis, is the following: red: sensitive, green: tolerant, black: variable.

The DEGs used and their respective regulation is described in Carvalho et al. (2017). In Figure 1, the distribution of expression of down-regulated (A) and up-regulated (B) DEGs is represented by whisker–box plots, for the clones analyzed. The classification through SLT analysis of tolerance/sensitivity is indicated by the clone’s code in green (tolerant), red (sensitive), or black (variable). In all clones, the expression of down- and up-regulated genes was consistent with their expected regulation tendency. Thus, this global gene analysis confirmed the expected regulation of the markers, as a whole. However, it is possible to verify that the stability of expression of both down-regulated and up-regulated genes was higher in sensitive and “variable” clones than in tolerant ones.

**FIGURE 2:**
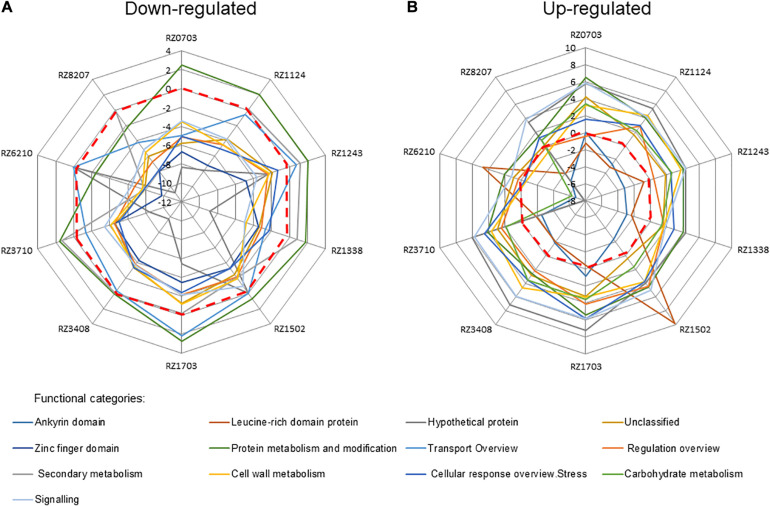
Relative relevance of functional categories of down-regulated **(A)** and up-regulated **(B)** DEGs (as in Supplementary Table 6) in 10 chosen Aragonez clones, by MapMan analysis. Values correspond to the average log_2__(gene expression ratio)_ of all DEGs in the mentioned functional category.

When allocating the significantly regulated DEGs in each clone to functional categories, some changes in regulation emerged (Figure 2); namely, the down-regulated category protein metabolism and modification became up-regulated in all clones but RZ8207, whereas secondary metabolism was up-regulated in all but RZ0703 and RZ6210 (Figure 2A). Conversely, the expected up-regulated ankyrin domain was down-regulated in all clones but RZ1703 and RZ0703, whereas leucine domain was down-regulated in all clones but RZ1502 and RZ6210 (Figure 2B). With these exceptions, all other functional categories followed the expected regulation in all clones.

**FIGURE 3:**
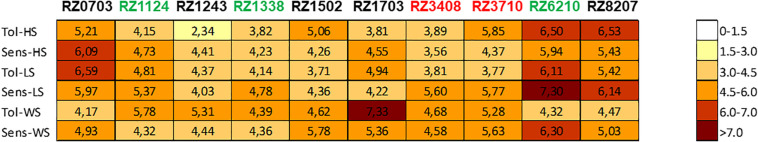
Stress matrix built using the average of the | log_2__(gene expression ratio)_| of HS/LS/WS DEGs (as in Supplementary Table 6) that yielded a significant value (| >1.5|) of the expected regulation (down-/up-). Expression in relation to Tol, tolerant; Sens, sensitive; HS, heat stress; LS, light stress; WS, water stress.

Chosen clones were ranked as sensitive/tolerant/variable to abiotic stress in the field by using SLT analysis. The gene expression of the array in the chosen 10 clones was analyzed relative to individual stresses (heat, light, and water stress), using the values of expression of the chosen up- and down-regulated DEGs that were associated with each stress (see Carvalho et al., 2017). The results obtained were compared with those of the reference tolerant variety (TN) and the reference sensitive variety (TR) as described in Carvalho et al. (2017). Thus, a “stress matrix” for light, heat, and water stress was obtained (Figure 3). The clones studied were subjected to high levels of stress, as described in Section “Materials and Methods” and consistent with a typical Mediterranean summer. As a result, there was no irresponsive clone in DEG expression analysis (only one value of expression lower than 3-fold was observed in the variable clone RZ1243 in the response to heat stress).

On the whole, RZ0703, RZ6210, and RZ8207 were the clones that showed higher increases of expression of the stress-associated DEGs. In the season 2015, the variable response clones RZ0703 and RZ8207 ranked as tolerant through analysis of PGV of SLT, and thus, this response was consistent with SLT ranking. The genotype RZ1243 was the one with a lower level of response to stress. Sensitive ranked clones through PGV of SLT analysis were also classified as weakly responsive to stress by the stress matrix (RZ3408 and RZ3710).

### Correlation Between DEG Stress Markers and SLT

**FIGURE 4:**
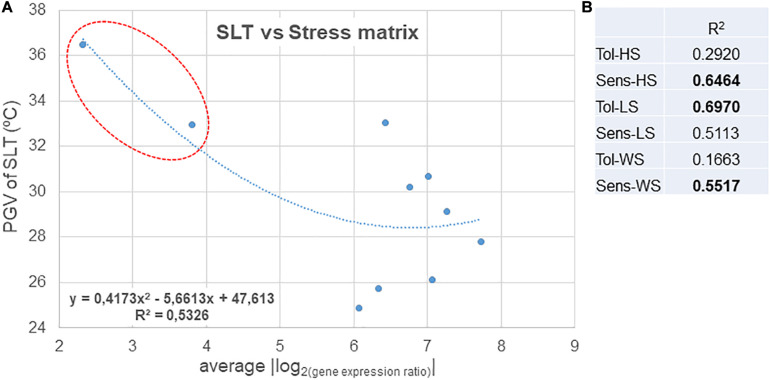
**(A)** Correlation between the stress matrix values and SLT values measured at the same time as the sampling for gene expression analysis. **(B)** Coefficient of determination for the correlations between SLT values and the stress matrix values for individual stresses in the tolerant and sensitive controls. Tol, tolerant; Sens, sensitive; HS, heat stress; LS, light stress; WS, water stress.

Gene expression was quantified in samples taken on the season 2015; therefore, the correlation between values obtained for the stress matrix was compared with PGV of SLT only of the 2015 analysis (Figure 4). The correlation with the overall stress indicators is quite high. When correlating SLT values with the individual stress markers, it was possible to find better correlations with the “sensitive” markers for heat and water stress and with the “tolerant” only for light stress, an indication that, overall, the variety Aragonez is fairly sensitive to heat and water stress and tolerant to excess light (Figure 4B). Also, the clones with the lowest PGV of SLT in 2015 (RZ0703 and RZ6210) are also the ones ranking as tolerant in the stress matrix indicators. Ranking fairly well in these indicators is clone RZ8207, which does not rank as good in overall SLT analysis because it is prone to high genotype × environment interaction. Nevertheless, in the specific season analysis, it was moderately tolerant, thus justifying a high level of response to stress, as seen by the stress matrix result (Figure 3).

**FIGURE 5:**
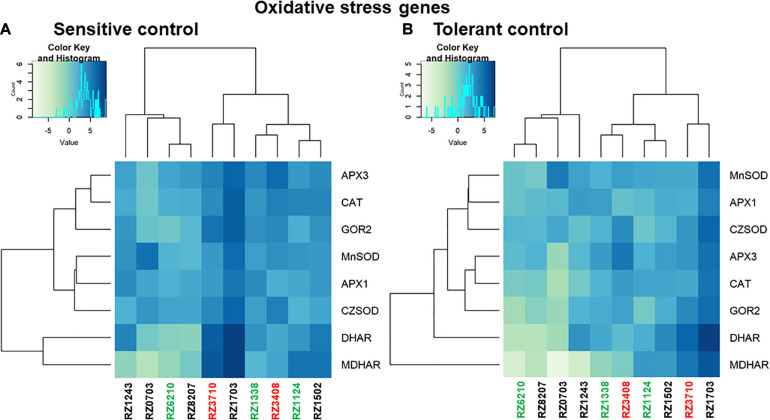
Relative gene expression ratios obtained by reverse transcriptase–qPCR of antioxidative stress response genes (as in Supplementary Table 6) in relation to control plants of the sensitive control **(A)** and the tolerant control **(B)**. Values were normalized with respect to *translation initiation factor eIF-3 subunit 4 (TIF)*, *translation initiation factor eIF-2B alpha subunit* (*TIF-GTP*) and *actin 2 (act)* mRNA. The data correspond to log_2__(gene expression ratio)_ of three independent samples measured in duplicate.

**FIGURE 6:**
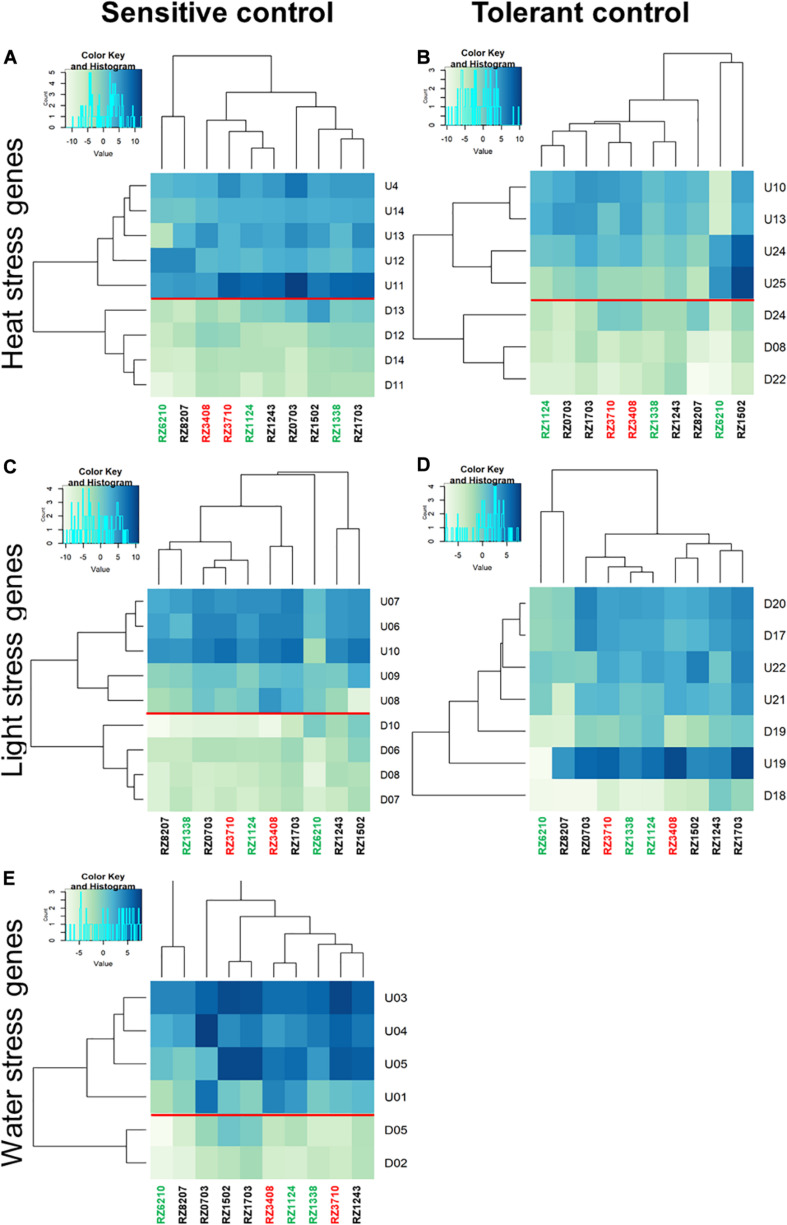
Relative gene expression ratios obtained by reverse transcriptase–qPCR of the stress matrix DEGs (as in Supplementary Table 6) quantified in the 10 chosen Aragonez clones. Relative expressions in relation to control plants of the sensitive control (left) and the tolerant control (right) were calculated for each individual stress (heat: **A,B**; light: **C,D**; and water: **E**). Values were normalized with respect to *translation initiation factor eIF-3 subunit 4 (TIF)*, *translation initiation factor eIF-2B alpha subunit* (*TIF-GTP*) and *actin 2 (act)* mRNA. The data correspond to log_2__(gene expression ratio)_ of three independent samples measured in duplicate.

Relative gene expression ratios were calculated in relation to control plants of the sensitive control (left) and the tolerant control (right) for antioxidative stress response DEGs (Figure 5), and for DEGs characterizing each individual stress (Heat, Light, and Water), as in Carvalho et al. (2017) (Figure 6). In Figures 5, 6, individual DEG expression of genes that were expected to be up- or down-regulated according to each individual stress is shown (as in Supplementary Table 6). The levels of individual expression of the array of stress DEGs correlated better with the expected patterns in the sensitive control than in the tolerant one. This can be explained because the variety Aragonez is more similar in its response to stress to TR (the sensitive control) than to TN (the tolerant one). In fact, water stress in the tolerant control represented a challenge, as some DEGs had to be removed (Carvalho et al., 2017). The expression of the remaining ones was quantified, but clustering was not significant with so few genes, and therefore, only the comparison with the sensitive control is shown for water stress (Figure 6E). The expression of up- and down-regulated DEGs clustered with the expected patterns in all clones and the levels of expression allowed the characterization of the clones regarding individual abiotic stress: the overall tolerant RZ6204 emerged as tolerant to heat and drought and moderately sensitive to light and all individual stresses; the variable clone RZ8207 was tolerant to all individual stresses in the season of 2015; and RZ0703, another variable clone but tolerant in 2015 through PGV of SLT analysis was tolerant to water and light and moderately sensitive to heat. The stress that affected the sensitive genotypes RZ3408 and RZ3710 to a larger extent was drought.

## Discussion

Currently, as climate changes impose a new order into Mediterranean vegetation, high-value crops begin feeling the pressure to move to higher latitudes and altitudes. However, crops whose economic revenue is closely linked with region, soil type, and characteristic genotypes are not so easy to move. One such species is grapevine, with high-value wine production closely associated with the *terroir* concept and also being subject to very strict region-specific regulations. Thus, it is not possible to change the geographical location of many high-value varieties to accommodate climate conditions. In Portugal, grapevine selection has evolved enormously in the last decades, with innovative methods developed that help increase quality and productivity in ancient varieties while at the same time preserving genetic variability (Martins and Gonçalves, 2015). This careful and exhaustive search for as many as possible different genotypes within each variety has led to the gathering of raw material for selection based on any desirable trait, provided that the adequate experimental setup is used in the establishment of the trials and that the right tools to scan an enormous amount of plant material in the field are available.

### Clonal Selection for Abiotic Stress

With this in mind, we set up to establish a non-destructive, easy-to-use, reproducible, and fast method to scan a grapevine selection experimental field for abiotic stress. The method chosen was the measurement of SLT. This parameter is highly influenced by the environment; thus, to obtain accurate and reproductive results, measurements must be made in an experimental field with a specific layout, in days with specific light, temperature, and wind conditions and in the hottest hours of the day and following a carefully established order. In fact, only an efficient experimental design allows to control the effects of the environment and, most importantly, to quantify the contribution of the genetic component through broad sense heritability and EBLUPs of genotypic effects. This process is time- and space-consuming and expensive. Hundreds of genotypes under evaluation in several repetitions occupy a large area (usually between 1.0 and 2.0 ha); an efficient control of the field installation cannot use ready-made grafted plants, and evaluations can only begin after at least 4 years of field occupation. Additionally, repetitions in different seasons must also be made to assess the genotype × environment interaction. Therefore, trials such as these are only feasible for economically important and traditional/regional varieties. Nevertheless, they are of paramount relevance for the preservation and evaluation of the intravarietal diversity of those varieties.

In this study, sources of variation such as seasonal fluctuations in environmental conditions put in evidence the importance of the adopted experimental design and the need to account for those variations in the model for SLT data analysis. In the results of the fitted model, the significant effects of the complete blocks reflect the control of spatial variation among complete blocks and the differences in air temperatures among the days of the measurements; the significant variance of incomplete blocks within complete block reveals the importance of taking into account the differences in air temperatures in each measurement interval (accounting for the time of day of the measurements); the significant variance of leaf within plot is associated with the control of the differences among plants in the same plot and to the exposition deviations of the measured leaves.

Results showed that there was significant genetic variability within the variety for SLT in the three seasons. However, in the season with moderate stress (2014, average Ψ_*pd*_ = −0.55), the range of values obtained for the EBLUPs of genotypic effects for SLT between sensitive and tolerant genotypes was lower than in the two seasons with severe stress. This was also reflected by the lower value obtained for broad sense heritability in 2014.

Although genetic variability among clones was found for SLT in all evaluated years, there were clones that maintained the same tendency for lower or higher SLT over the years, whereas others had more unstable performance. The latter ones correspond to genotypes that are more sensitive to genotype × environment interaction. This phenomenon occurs for any quantitative trait, as is the case of SLT. Therefore, when selecting clones, an important criterion is the selection of those with lower sensitivity to genotype × environment interaction for the evaluated traits. For this reason, the classification of clones selected in just 1 year (2014) for gene expression analysis was changed when performing the final selection of genotypes, in which the sensitivity to genotype × environment interaction was also taken into account.

### Integration of the Evaluated Traits

As the selection of genotypes of well-established high-value varieties only makes sense in the context of high-quality and good levels of production, genotypes were also monitored for quality of the must, even if those genotypes had already been subject to selection for those traits (Gonçalves and Martins, 2019). Quality traits showed the existence of significant genetic variability (for any usual significance level). For acidity, anthocyanins, total phenols, and berry weight, the values obtained for heritability were similar to those obtained for SLT. This represents a relevant result for SLT because, theoretically, this trait should be subject to higher environmental variability than anthocyanins, total phenols, and berry weight. Therefore, such result confirms that a good experimental design and an adequate model were applied to study SLT. Also, according to previous works (Gonçalves et al., 2013), higher estimates for heritability were consistently obtained when using the average of years because, for each clone, a convergence of the phenotypic values to the true genotypic value is expected.

Genetic correlations between SLT and quality traits estimated for each season were approximately zero. This result indicates that, when exploring the genetic variability within the variety, several genotypes satisfying several criteria, for example, simultaneously more tolerant to stress and with quality traits above the mean of the population studied, can be selected. This indicates that quality will not be lost when selecting for stress tolerance. In general, the low genetic correlations observed between traits are in agreement with the findings of previous studies on selection in grapevine ancient varieties (Gonçalves et al., 2016).

The DEGs used in this analysis were described as being able to characterize tolerance/sensitivity to stress in grapevine varieties (Carvalho et al., 2017). Because of the enormous plasticity of the grapevine transcriptome (Dal Santo et al., 2013) and the fact that Aragonez is a variety with high intravarietal variability (Gonçalves et al., 2007; Gonçalves and Martins, 2019), it was expected that differences in gene expression between the clones of the experiment would emerge. In fact, the analysis of the “stress array” identified the clones as sensitive or tolerant to stress, in the same pattern as the SLT analysis of that single season. However, SLT analysis in successive years showed that some of those initially regarded as tolerant or sensitive in fact belonged to a yet unidentified group of highly variable genotypes with contradictory results in successive years. It must be emphasized that gene expression analysis of these clones in one single year could yield false results, as they will respond differently from season to season.

### Physiological Aspects of Tolerance/Sensitivity

The foundation for SLT analysis is that a plant that can keep its leaf temperature lower will only be able to do so if it has a good control of stomatal opening and thus of its transpiration. This will be associated with a better modulation of gas exchange and thus higher levels of CO_2_ uptake. These plants will be able to produce more photoassimilates that will be available for berry production. These conditions during the plant’s life cycle contribute to better growth rates, higher level of photoassimilate storage for winter and thus higher number of productive flowers that will lead to higher yield in the following season. In fact, as a predicted genetic gain of yield of +14.1% was observed, this tendency of more tolerant genotypes to show a yield performance above the mean of the population was confirmed.

Oxidative stress response genes were not present in the original array (Carvalho et al., 2017), but as they are key players in abiotic stress response in general and specifically in grapevine (Terrier et al., 2005; Carvalho et al., 2015a), they were included in this study. All the genes were up-regulated, especially in RZ1703 and RZ3710, an indication of these clones’ sensitivity to stress. The three clones that behaved as tolerant in the year chosen, RZ6210, RZ8207, and RZ0703, were the less affected by oxidative stress. In these clones, hydrogen peroxide scavenging through CAT and APX was predominant, while in more sensitive genotypes, the whole asc-glut cycle was up-regulated, with higher levels of expression of GOR and DHAR (for review, see Carvalho et al., 2015b). Regarding the comparison with the individual stress markers, all the Aragonez clones studied clustered better with the sensitive control in all stresses, confirming previous results of sensitivity to water stress (Martorell et al., 2015) in the variety as a whole.

## Conclusion

It was possible to identify intravarietal genetic variability for the several traits analyzed in the field trial of Aragonez accessions, including for SLT. This variation could be useful in the improvement of genotypes of this variety to withstand stress conditions and to better adapt to climate change.

To sum up, the selection of a group of genotypes tolerant to abiotic stress was performed, with an increase of the mean yield and maintaining the behavior for quality traits.

## Data Availability Statement

The original contributions generated for this study are included in the article/Supplementary Materials, further inquiries can be directed to the corresponding author.

## Author Contributions

EG, LC, and AM conceived the ideas and the overall study design. LC and EG designed the methodology and collected the data. LC and EG analyzed the data. LC and EG wrote the manuscript. SA and AM revised it. All authors contributed with reagents, materials, and analysis tools. All authors contributed critically to the drafts and gave final approval for publication.

## Conflict of Interest

The authors declare that the research was conducted in the absence of any commercial or financial relationships that could be construed as a potential conflict of interest.

## References

[B1] Butler D. G. Cullis B. R. Gilmour A. R. Gogel B. J. Thompson R. (2018). *ASReml-R Reference Manual, Version 4.* Wollongong, NSW: University of Wollongong.

[B2] Carbonneau A. Champagnol F. (1993). *Nouveaux Systemes de Culture Integre du Vignoble. Programme AIR-3-CT.* 93.

[B3] Carvalho L. C. Coito J. L. Colaço S. Sangiogo M. Amâncio S. (2015a). Heat stress in grapevine: the pros and cons of acclimation. *Plant Cell Environ.* 38 777–789. 10.1111/pce.12445 25211707

[B4] Carvalho L. C. Coito J. L. Gonçalves E. F. Chaves M. M. Amâncio S. (2016). *Plant Biol.* 18 101–111. 10.1111/plb.12410 26518605

[B5] Carvalho L. C. Silva M. Coito J. L. Rocheta M. P. Amâncio S. (2017). Design of a custom RT-qPCR array for assignment of abiotic stress tolerance in traditional Portuguese grapevine varieties. *Front. Plant Sci.* 8:1835. 10.3389/fpls.2017.01835 29118776 PMC5660995

[B6] Carvalho L. C. Vidigal P. Amâncio S. (2015b). Oxidative stress mediated signaling and homeostasis in grapevine (*Vitis vinifera* L.). *Front. Environ. Sci.* 3:20 10.3389/fenvs.2015.00020

[B7] Chaves M. M. Zarrouk O. Francisco R. Costa J. M. Santos T. Regalado A. P. (2010). Grapevine under deficit irrigation: hints from physiological and molecular data. *Ann. Bot.* 105 661–676. 10.1093/aob/mcq030 20299345 PMC2859908

[B8] Coito J. Rocheta R. Carvalho L. C. Amâncio S. (2012). Microarray-based uncovering reference genes for quantitative real time PCR in grapevine under abiotic stress. *BMC Res. Notes* 5:220. 10.1186/1756-0500-5-220 22564373 PMC3837474

[B9] Dal Santo S. Tornielli G. B. Zenoni S. Fasoli M. Farina L. Anesi A. (2013). The plasticity of the grapevine berry transcriptome. *Genome Biol.* 14:r54. 10.1186/gb-2013-14-6-r54 23759170 PMC3706941

[B10] Degu A. Hochberg U. Sikron N. Venturini L. Buson G. Ghan R. (2014). Metabolite and transcript profiling of berry skin during fruit development elucidates differential regulation between Cabernet Sauvignon and Shiraz cultivars at branching points in the polyphenol pathway. *BMC Plant Biol.* 14:188. 10.1186/s12870-014-0188-4 25064275 PMC4222437

[B11] Deloire A. Carbonneau A. Wang Z. Ojeda H. (2004). Vine and water, a short review. *J. Int. Sci. Vigne Vin* 38 1–13. 10.20870/oeno-one.2004.38.1.93218

[B12] Downey M. O. Dokoozlian N. K. Krstic M. P. (2006). Cultural practice and environmental impacts on the flavonoid composition of grapes and wine: a review of recent research. *Am. J. Enol. Vitic.* 57 257–268.

[B13] Dry P. R. Loveys B. R. Düring H. (2000). Partial drying of the rootzone of grape. II. Changes in the pattern of root development. *Vitis* 39 9–12.

[B14] Fuchs M. (1990). Infrared measurement of canopy temperature and detection of plant water stress. *Theor. Appl. Climatol.* 42 253–261. 10.1007/BF00865986

[B15] Gilmour A. Thompson R. Cullis B. (1995). Average information REML: an efficient algorithm for variance parameter estimation in linear mixed models. *Biometrics* 51 1440–1450. 10.2307/2533274

[B16] Gonçalves E. Carrasquinho I. Almeida R. Pedroso V. Martins A. (2016). Genetic correlations in grapevine and their effects on selection. *Aust. J. Grape Wine Res.* 22 52–63. 10.1111/ajgw.12164

[B17] Gonçalves E. Carrasquinho I. St. Aubyn A. Martins A. (2013). Broad-sense heritability in the context of mixed models for grapevine initial selection trials. *Euphytica* 189 379–391. 10.1007/s10681-012-0787-9

[B18] Gonçalves E. Martins A. (2019). Genetic gains of selection in ancient grapevine cultivars. *Acta Hortic.* 1248 47–54. 10.17660/ActaHortic.2019.1248.7

[B19] Gonçalves E. St. Aubyn A. Martins A. (2007). Mixed spatial models for data analysis of yield on large grapevine selection field trials. *Theor. Appl. Genet.* 115 653–663. 10.1007/s00122-007-0596-z 17665169

[B20] Gonçalves E. St. Aubyn A. Martins A. (2010). Experimental designs for evaluation of genetic variability and selection of ancient grapevine varieties: a simulation study. *Heredity* 104 552–562. 10.1038/hdy.2009.153 19904297

[B21] Gonzalez-Dugo V. Durand J.-L. Gastal F. (2010). Water deficit and nitrogen nutrition of crops. A review. *Agron. Sustain. Dev.* 30 529–544. 10.1051/agro/2009059

[B22] Henderson C. (1975). Best linear unbiased estimation and prediction under a selection model. *Biometrics* 31 423–447. 10.2307/2529430 1174616

[B23] International Organisation of Vine and Wine [OIV]. (2018). *World Vitiviniculture Situation OIV Statistical Report on World Vitiviniculture.* Paris: OIV.

[B24] Lovisolo C. Perrone I. Carra A. Ferrandino A. Flexas J. Medrano H. (2010). Drought-induced changes in development and function of grapevine (*Vitis* spp.) organs and in their hydraulic and non-hydraulic interactions at the whole-plant level: a physiological update. *Func. Plant Biol.* 37 98–116. 10.1071/FP09191

[B25] Martins A. Gonçalves E. (2015). “Grapevine breeding programmes in Portugal,” in *Grapevine Breeding Programs for the Wine Industry: Traditional and Molecular Techniques*, ed. Reynolds A. G. (Sawston: Woodhead Publishing), 159–182. 10.1016/b978-1-78242-075-0.00008-9

[B26] Martorell S. Diaz-Espejo A. Tomàs M. Pou A. El Aou-ouad H. Escalona J. M. (2015). Differences in water-use-efficiency between two *Vitis vinifera* cultivars (Grenache and Tempranillo) explained by the combined response of stomata to hydraulic and chemical signals during water stress. *Agric. Water Manag.* 156 1–9. 10.1016/j.agwat.2015.03.011

[B27] Parker A. de Cortazar Atauri G. van Leeuwen C. Chuine I. (2011). General phenological model to characterise the timing of flowering and véraison of *Vitis vinifera* L. *Aust. J. Grape Wine Res.* 17 206–216. 10.1111/j.1755-0238.2011.00140.x

[B28] Patterson H. D. Thompson R. (1971). Recovery of inter-block information when block sizes are unequal. *Biometrika* 58 545–554. 10.2307/2334389

[B29] R Core Team (2018). *R: A Language and Environment for Statistical Computing.* Vienna: R Foundation for Statistical Computing.

[B30] Resolution OIV-VITI 564B-2019. (2019). OIV Process for the Recovery and Conservation of the Intra-varietal Diversity and the Polyclonal Selection in Grape Varieties with Wide Genetic Variability. Available online at: http://www.oiv.int/en/technical-standards-and-documents/resolutions-of-the-oiv/viticulture-resolutions

[B31] Rocheta M. Coito J. L. Ramos M. Carvalho L. C. Becker J. D. Carbonell-Bejerano P. (2016). Transcriptomic comparison between two *Vitis vinifera* L. varieties (Trincadeira and Touriga Nacional) in abiotic stress conditions. *BMC Plant Biol.* 2016:224. 10.1186/s12870-016-0911-4 27733112 PMC5062933

[B32] Schultz H. (2003). Differences in hydraulic architecture account for near-isohydric and anisohydric behaviour of two field-grown *Vitis vinifera* L. cultivars during drought. *Plant Cell Environ.* 26 1393–1405. 10.1046/j.1365-3040.2003.01064.x

[B33] Self S. G. Liang K. Y. (1987). Asymptotic properties of maximum likelihood estimators and likelihood ratio tests under nonstandard conditions. *J. Am. Stat. Assoc.* 82 605–610. 10.1080/01621459.1987.10478472

[B34] Somers T. C. Evans M. E. (1977). Spectral evaluation of young red wines: anthocyanin equilibria, total phenolics, free and molecular so2, “chemical age”. *J. Sci. Food Agric.* 28 279–287. 10.1002/jsfa.2740280311

[B35] Stram D. O. Lee J. W. (1994). Variance components testing in the longitudinal mixed effects model. *Biometrics* 50 1171–1177. 10.2307/2533455 7786999

[B36] Terrier N. Glissant D. Grimplet J. Barrieu F. Abbal P. Couture C. (2005). Isogene specific oligo arrays reveal multifaceted changes in gene expression during grape berry (*Vitis vinifera* L.) development. *Planta* 222 832–847. 10.1007/s00425-005-0017-y 16151847

[B37] Udompetaikul V. Upadhyaya S. K. Slaughter D. Lampinen B. Shackel K. (2011). *Plant Water Stress Detection Using Leaf Temperature and Microclimatic Information.* ASABE Paper No 1111555 St. Joseph, MI: ASABE, 10.13031/trans.57.10319

[B38] van Leeuwen C. Darriet P. (2016). The impact of climate change on viticulture and wine quality. *J. Wine Econ.* 11 150–167. 10.1017/jwe.2015.21

[B39] van Leeuwen C. Trégoat O. Choné X. Bois B. Pernet D. Gaudillère J.-P. (2009). Vine water status is a key factor in grape ripening and vintage quality for red Bordeaux wine. How can it be assessed for vineyard management purposes? *J. Int. Sci. Vigne Vin* 43 121–134. 10.20870/oeno-one.2009.43.3.798

[B40] Vandeleur R. K. Mayo G. Shelden M. C. Gilliham M. Kaiser B. N. Tyerman S. D. (2009). The role of plasma membrane intrinsic protein aquaporins in water transport through roots: diurnal and drought stress responses reveal different strategies between isohydric and anisohydric cultivars of grapevine. *Plant Physiol.* 149 445–460. 10.1104/pp.108.128645 18987216 PMC2613730

[B41] Zarrouk O. Garcia-Tejero I. Pinto C. Genebra T. Sabir F. Prista C. (2016). Aquaporins isoforms in cv. Touriga Nacional grapevine under water stress and recovery—regulation of expression in leaves and roots. *Agric. Water Manag.* 164 167–175. 10.1016/j.agwat.2015.08.013

